# SHBG and Insulin resistance - Nexus revisited

**DOI:** 10.6026/973206300200816

**Published:** 2024-08-31

**Authors:** Asha Dinakaran, Srinivasan AR, Reeta Rajagambeeram, Sunil Kumar Nanda, Mary Daniel

**Affiliations:** 1 Department of Biochemistry, Pondicherry Institute of Medical Sciences, Kalapet, Pondicherry, India; 2 Department of Biochemistry, Mahatma Gandhi Medical College & Research Institute, Sri Balaji Vidyapeeth, Deemed to be University, Pillaiyarkuppam, Pondicherry, India; 3 Department of Obstetrics & Gynaecology, Pondicherry Institute of Medical Sciences, Kalapet, Pondicherry, India

**Keywords:** SHBG, IR, SNP

## Abstract

Sex hormone binding globulin (SHBG) is a liver-synthesized glycoprotein. Low SHBG levels are associated with insulin resistance (IR). Specific single nucleotide polymorphisms (SNPs) in the SHBG gene are linked to IR. Therefore, it is of interest to provide a review on the comprehensive overview for SHBG related to IR.

## Background:

The primary role of Sex Hormone Binding Globulin (SHBG) has traditionally been viewed as that of a transport protein for sex steroids, regulating circulating concentrations of free (unbound) hormones and their transport to target tissues [1]. The extracellular levels of SHBG are considered important in the regulation of plasma-free and albumin-bound sex steroids (androgens and estrogens) [2]. There is an inverse relationship between serum insulin and SHBG, with low levels of SHBG being associated with IR [3]. Studies have explored the role of SHBG in type 1 diabetes mellitus (T1DM) [4] and its predictive value in gestational diabetes mellitus (GDM) [5]. Several SNPs in the SHBG gene have been identified, with SNPs rs6257 and rs6259 in exon 2 being linked to IR [6]. Therefore, it is of interest to report that understanding SHBG, its receptor, and related SNPs could lead to new therapeutic approaches in precision medicine for managing IR, T2DM, and GDM.

## SHBG:

SHBG is a 90 kDa homo dimeric glycoprotein, composed of two subunits of 373 amino acid residues and has high binding affinity for testosterone and dihydrotestosterone (DHT) and lower affinity for 17-beta oestradiol. Each SHBG protein has two steroid-binding sites, which can be unoccupied, partially occupied, or fully occupied at any time. Different sex steroids can simultaneously bind to the same SHBG protein. When a ligand or agonist binds, it induces conformational changes in the hormone-binding pocket of the SHBG protein. SHBG is believed to have specific oligosaccharide side chains, which may be necessary for the SHBG-steroid complex to interact with receptors on the cell surface of target tissues [7]. The SHBG gene, which encodes both plasma SHBG and testicular androgen-binding protein synthesized in Sertoli cells, consists of eight exons within a 4-kb segment. This gene is located on the short arm of chromosome 17 (p12-p13) [8]. The biosynthesis of SHBG involves the activation of the SHBG promoter in the liver, which requires the nuclear factor hepatocyte nuclear factor 4 alpha (HNF4A). This factor binds to a DR1-like cis element, leading to the production of SHBG [9]. The metabolic clearance of SHBG from the bloodstream occurs in two phases where the initial phase happens over a few hours, followed by a longer phase with a half-life of several days. Research indicates that the degree of glycosylation directly affects plasma clearance [3]. In humans, plasma SHBG levels are tightly regulated by both endocrine and non-endocrine factors. It is documented that exogenous estrogens and thyroid hormone significantly increase plasma SHBG levels, whereas anabolic androgens and androgenic progestins decrease them [10]. Low-plasma SHBG levels are found in obese individuals and have been shown to be associated with an increased risk of diabetes mellitus (DM) and related complications, including cardiovascular disorders [11]. SHBG is frequently correlated positively with high-density lipoprotein (HDL) cholesterol and negatively with triacylglycerol's and insulin [16]. A positive correlation between SHBG levels and various measures of insulin sensitivity has been documented, regardless of gender. This suggests that reduced SHBG levels may be a significant factor in the development of metabolic syndrome, alongside traditional factors such as hypertension, abdominal obesity, and hyperinsulinism.

## Molecular mechanism of action of SHBG:

The receptor for SHBG operates through a G-protein-coupled receptor mechanism. 12 When SHBG binds to its specific receptor; it activates the G-protein-coupled second messenger system, resulting in the intracellular release of cyclic adenosine monophosphate (cAMP). This activation then triggers protein kinase A (PKA) [13]. Experimental evidence shows that SHBG membrane binding decreases in the presence of a non-hydrolyzable analogue of guanosine triphosphate. Additionally, when the alpha-subunit of the G-protein is replaced with a mutant form, the intracellular rise in cAMP does not occur [14]. The binding of different sex steroid ligands to SHBG results in varying changes in the intracellular second messenger system. Estradiol acts as an agonist, testosterone as an antagonist, and the active metabolite DHT can act as either an agonist or antagonist depending on the tissue-specific location of the receptor [15]. This signaling system may also influence the expression of androgen or estrogen receptors. These observations highlight the dynamic presence of a SHBG plasma membrane receptor and an intricate second messenger system, underscoring the extra-genomic actions of androgens and estrogens [16].

## Quantitation of SHBG and reference levels:

Currently, both Immunoradiometric and mass spectrometry-based assays are available for estimating SHBG [17]. Monoclonal antibody-based assays are presently acquiring relevance. Binding assays for SHBG have been developed wherein SHBG is saturated with tritiated DHT [18]. In addition, ELISA-based kits are also available, but frequently such assays are utilised for research purposes, rather than for routine determination in serum or plasma [19].

The normal (reference) range for SHBG concentrations in adults as follows:

[1] Male: 10-57 nmol/L

[2] Female (non-pregnant): 18-144 nmol/L

[3] The reference range would vary with the nature and type of assay method and hence inter-laboratory variations are to be taken while interpreting the laboratory results [20].

Men have lower SHBG levels than women. In aging men, the levels of SHBG are on the higher side, which is attributed to declining testosterone levels. Pregnancy enhances SHBG levels, which return to basal levels following childbirth [21].

## Salient features of insulin action and resistance:

Insulin, a peptide hormone is produced by the beta cells of islets of Langerhans. Insulin is the only hormone in the body responsible for reducing blood sugar levels. Following its release into the circulation, it reaches the insulin sensitive tissues, namely adipose tissue, skeletal muscle and cardiac muscle [22]. The insulin-regulated glucose transporter 4 GLUT4 is in the adipose tissues, as well as the skeletal and cardiac muscle. GLUT4 plays a crucial role in transporting glucose from the blood into tissues, which occurs primarily after insulin binds to its specific receptor on the cell membrane [23]. This insulin receptor, a member of the tyrosine kinase superfamily, has two subunits [24]. Upon activation, the receptor triggers a complex intracellular signaling network mediated by insulin receptor substrate (IRS) proteins and the canonical phosphatidylinositol 3-kinase (PI3K) and extracellular signal-regulated kinase (ERK) pathways [25]. Consequently, the two main insulin signaling pathways that originate from the insulin receptor-IRS complex are the PI3K/AKT (also known as protein kinase B) pathway and the Raf/Ras/MEK/MAPK (also known as ERK) pathway [26]. IR is a complex phenomenon that is characterised by long-term complications involving cardiovascular, renal and ocular systems, in addition to the peripheral nervous system. IR involves poorly functioning adipose tissue and disrupted insulin signaling due to lipotoxicity, which leads to a triad of glucotoxicity, oxidative stress, and low-grade inflammation [27]. The defective insulin-stimulated glucose transport activity is primarily due to increased lipid metabolites in muscle cells. These metabolites, such as acyl CoAs and diacylglycerol, activate a serine/threonine kinase cascade that impairs insulin signaling by phosphorylating IRS-1 on serine/threonine residues [28]. This process is a major mechanism implicated in IR.

Although established evidence highlights the complications of IR in obese individuals and those with type 2 diabetes, new approaches to diabetes subtyping are offering fresh insights. This subtyping, also known as clustering has revealed varying levels and durations of IR among people with diabetes. One specific subtype, termed severe insulin resistant diabetes (SIRD), exhibits significant metabolic abnormalities and indicates a risk for cardiovascular, renal, and hepatic comorbidities [29]. In recent years, exosomes have been associated with IR. These small (nano-sized) bio-vesicles are released into bodily fluids following the fusion of multivesicular bodies with the cell membrane [30]. Exosomes carry cell-specific lipids, proteins, and genetic materials. They can be selectively absorbed by nearby or distant cells, ultimately leading to the reprogramming of the recipient cells. Recent research on IR highlights that, in addition to metabolites and signaling proteins, exosomes carrying proteins, mRNA, and microRNA significantly influence the molecular interactions within insulin-sensitive tissues such as adipose tissue and skeletal muscle. Additionally, exosomes impact the liver, the central metabolic clearing house. These processes are associated with the onset and progression of IR [31]. The typical molecular mechanisms underlying IR, including the increasing role of exosomes, are strong candidates in the management of IR and thus acquire a relevant role in personalized medicine.

## SHBG and IR:

The association between SHBG and metabolic syndrome implies a connection with IR [32]. Although IR becomes significant over the course of diabetes, SHBG levels are inversely related to glycated hemoglobin (HbA1c), in normal individuals. This suggests that SHBG is related to glucose homeostasis even before the onset of DM. Low levels of total testosterone and SHBG were significantly linked to developing metabolic syndrome, independent of cardiovascular risk factors and IR [33]. Testosterone might turn out to be a key player in controlling insulin sensitivity in men. The levels of testosterone are low in men with T2DM and coronary heart diseases indicating that testosterone replacement therapy could potentially improve insulin sensitivity in men [34]. Hence, SHBG levels are implicated under such conditions. The waist-to-hip ratio (WHR) is linked to testosterone levels in men and associated with attenuation in glucose metabolism. This signifies the connection between SHBG and IR in relation to the characteristic anthropometric measurements [35]. Abdominal obesity is regarded as a key factor in suboptimal testosterone levels, regardless of diabetes status. Evidence suggests that the causal relationship between low testosterone and T2DM may be complex and interconnected with obesity and insulin resistance, which are core aspects of MetS [36]. The Study of Women's Health Across the Nation (SWAN) has deciphered a strong independent link between increased hepatic lipids and reduced SHBG levels which is linked to an increased metabolic risk in middle-aged women [37]. This shows the role of hepatic lipids in modulating the influence of SHBG on insulin levels [38]. It may be noted that the plasma levels of SHBG are lowered in women with polycystic ovarian syndrome, an insulin-resistant condition with vulnerability to frank T2DM [39]. Obesity affects the extracellular levels of endogenous sex steroids in postmenopausal women. SHBG concentrations are associated with key features of metabolic syndrome in these women. These findings suggest that SHBG could be a valuable indicator of IR in postmenopausal obese women [40].

Extracellular SHBG prove to be a determinant of cardiometabolic disorders, independent of abdominal obesity and IR. This study was conducted on elderly men and women. In both genders, it has been observed that low SHBG is linked to altered triglyceride/ HDL levels. It is also observed that low SHBG among women is linked to elevated apolipoprotein B. In view of these considerations, SHBG levels might prove to be an independent factor for cardiometabolic morbidity in women inflicted with IR [41].

Recent evidence highlights the role of SHBG in the development of GDM [42]. Women with GDM have defective receptors in their placental tissue that impair insulin signal transduction, along with malfunctioning GLUTs [43]. A decline in SHBG expression may be linked to the regulation of insulin signalling, reflecting a decrease in the expression of key insulin-signalling components. This scenario in placental tissue can lead to the onset of GDM. SHBG regulates GLUT1 expression through the cAMP/PKA/CREB1 pathway, impacting glucose transport in the placenta, which significantly contributes to IR and GDM [44]. Studies have documented that insulin-like growth factor-1 (IGF-1) could be the chief regulator of SHBG with Insulin through its interaction with the IGF-1 receptors could potentially inhibit SHBG activity [45].

A study between sex steroids, SHBG, leptin, and IR in obese men found that levels of total testosterone and SHBG were lower in the obese group in comparison to the non-obese and overweight groups. SHBG demonstrated a significant negative correlation with both BMI and IR. In addition, testosterone and SHBG concentrations dwindled with an increasing BMI and IR [46].

## Gene polymorphism of SHBG and its revelation:

Relationship with IR:

Though it is well documented that the measurements of SHBG are used to predict free androgen levels in patients afflicted by excess and over exuberant androgen exposure, it needs to be realized that broader utility rests with SHBG in assessing the risk of developing endocrine diseases and more specifically in the realms of MetS with concomitant IR.SHBG gene polymorphism (rs1799941) has been linked to MetS in children and adolescents [47]. SHBG gene polymorphisms, namely rs6257 (CC,CT) and rs6259 (GG), have been reported to be associated with a pronounced risk of T2DM and linked to low circulating levels of SHBG, based on the genotype analysis of the exon 2 of SHBG [48]. Studies conducted in China suggest that rs6259 SNP is associated with the risk of MetS and accompanying lower serum SHBG levels in males of Chinese Han origin [49]. However, gene polymorphisms of SHBG are also implicated in other conditions, including polycystic ovary syndrome, reduced bone mineral density and cancer of the breast and prostate. According to The Tromso Study the SNP, rs1799941, in the SHBG gene may contribute to myocardial infarction, T2DM [50].

## SHBG as a predictor of GDM:

Gestational diabetes mellitus (GDM) represents maternal pathology typified by maternal glucose intolerance during the period of pregnancy. Therefore, both the mother and offspring would suffer from morbidity. Metabolism during pregnancy is unique and is associated with a plethora of changes that might culminate in GDM. GDM is regarded as a heterogeneous disease whose etiology remains unclear. The fact remains that GDM is on the rise and more importantly associated with the present-day lifestyle. As a rule, the diagnosis of GDM is based on the guidelines laid down by the International Association of Diabetes and Pregnancy Study Groups [51]. An oral glucose tolerance test is usually performed between the 24th and 28th weeks of gestation. However, the diagnosis facilitated by this conventional method at this stage of pregnancy might not prove to be beneficial enough to protect the mother and her offspring from GDM. Therefore, there is an urgent need for GDM markers/predictors to enable prevention and intervention at an earlier stage [52]. It needs to be categorically stated that the various markers of GDM, including plasma glucose, insulin, C-peptide, homeostatic model assessment of IR, etc., could merely differentiate between healthy pregnant women and those with GDM. Unfortunately, such methods are of no avail in enabling the early diagnosis of GDM. Low SHBG levels before pregnancy are linked to a higher risk of GDM, which could assist in the identification of women at risk and implementing early prevention measures. Women with SHBG levels below the median and a BMI of +25.0 kg/m2 have a fivefold higher chance of developing GDM compared to normal-weight women with SHBG levels at or above the median [53]. Caglar *et al.* delineated the fact that a high predictive accuracy of SHBG in early pregnancy (13-16weeks) of GDM could warrant insulin therapy. Furthermore, subgroup analysis revealed that the predictive accuracy of SHBG remained consistent across different age groups and BMI categories [42, 54 & 55]. However, no significant difference in SHBG concentration was observed between individuals with normal glucose tolerance and those with abnormal glucose tolerance [56]. A plethora of gene polymorphisms have been identified that could predispose to GDM [57, 58]. The SHBG SNP rs6259 gene polymorphism affects alterations in placental SHBG concentration and could thus play a pivotal role in the molecular mechanisms signifying GDM [59]. A succinct, yet comprehensive profile of the myriad facets has been highlighted with reference to GDM [60], wherein the role of genetic factors associated with GDM was highlighted. This could gain importance in the light of early predictors of GDM.

## Personalized medicine/precision medicine in DM (Role of SHBG):

Personalized medicine pertains to the advanced nuances in internal medicine [61]. In this context, the comprehensive genetic profile of the individual is a prerequisite for making cardinal and decisive recommendations that could aid in the diagnosis, and prognosis of a given clinical condition. According to data from the UK Biobank, the presence of circulating SHBG has a profound impact on various physiological and biochemical aspects, as related to IR [62]. In an era of pharmacogenomics and personalized medicine, it is imperative to conduct evidence-based research aimed at understanding the specific biological moorings of SHBG. This approach could eventually culminate in the identification of targeted interventions with significant implications for personalized medicine, especially in addressing IR. Recent experimental investigations have highlighted the key finding that SHBG might be involved in regulating glucose metabolism through its influence on multiple GLUTs, thereby suggesting that SHBG gene variations could have a crucial role in the development of IR and gestational diabetes [63]. Further studies are required in the field of personalized medicine to fully elucidate the regulatory mechanisms of GLUTs modulated using SHBG.

## Conclusion:

SHBG is a potential target in the stratification of the risk management for DM and the mitigation of IR. Furthermore, genomic and phenotypic considerations of SHBG would provide novel insights into the aspects governing the early prediction of GDM, besides personalized medicine for treating IR, the major player in the complications of DM.
